# Myogenic IGFBP5 levels in rhabdomyosarcoma are nourished by mesenchymal stromal cells and regulate growth arrest and apoptosis

**DOI:** 10.1186/s12964-025-02171-6

**Published:** 2025-04-15

**Authors:** Yue Zhang, Karim Katkhada, Liu Zhen Meng, Binbin Zhao, Shanlin Tong, Wiem Chaabane, Aditi Kallai, Nicholas P. Tobin, Arne Östman, Alessandro Mega, Monika Ehnman

**Affiliations:** 1 Department of Oncology-Pathology, Karolinska Institutet, Stockholm, Sweden; 2 Breast Center, Karolinska Comprehensive Cancer Center, Karolinska University Hospital, Stockholm, Sweden; 3 Centre for Cancer Biomarkers CCBIO, University of Bergen, 5021 Bergen, Norway; 4 PO Bröst- och endokrina tumörer och sarkom, Tema Cancer, Karolinska University Hospital, Visionsgatan 4, SE-171 76 Stockholm, Sweden

**Keywords:** Rhabdomyosarcoma, Mesenchymal stromal cells, IGFBP5, Apoptosis, Growth arrest

## Abstract

**Background:**

Mesenchymal stromal cells belong to a diverse collection of cells in different states that are poorly characterized in soft-tissue sarcomas. In this study, we explored tumor growth-regulatory signaling between differentially educated non-malignant mesenchymal stromal cells and malignant cells in pediatric rhabdomyosarcoma (RMS).

**Methods:**

Xenograft experiments demonstrated that non-malignant stromal cells influence tumor behavior. Gene expression analysis identified deregulated genes, which were further studied using cell culture assays and patient data. Clinicopathological correlations were made in a discovery cohort (*N* = 147) and a validation cohort (*N* = 101).

**Results:**

The results revealed transiently suppressive paracrine effects of orthotopic stromal cells derived from skeletal muscle. These effects were lost when the stromal cells were exposed to RMS cells, either short-term in vitro, or long-term in hindlimb muscle in vivo. High resolution microarray-based Clariom D gene expression analysis identified insulin-like growth factor binding protein 5 (*IGFBP5*) as the top upregulated gene in RMS cells exposed to naïve stromal cells, and effects on growth arrest, caspase 3/7 activation, and myogenic cell identity were demonstrated in functional assays. Furthermore, *IGFBP5* associated with the caspase 3 substrate growth arrest specific protein 2 (*GAS2*), lower disease stage and favorable survival in patient cohorts.

**Conclusions:**

This study uses functional modeling and omics approaches to identify IGFBP5 as a candidate mediator of anti-tumor growth mechanisms originating from tumor-neighboring mesenchymal stromal cells. Tumors of mesenchymal origin, such as RMS, are known for their heterogeneity, and this could potentially pose a limitation to the study. However, a clinical relevance is emphasized by consistent findings across patient cohorts. These insights pave the way for novel therapeutic strategies modulating activities of stromal cell subsets at primary and metastatic sites in RMS.

**Graphical Abstract:**

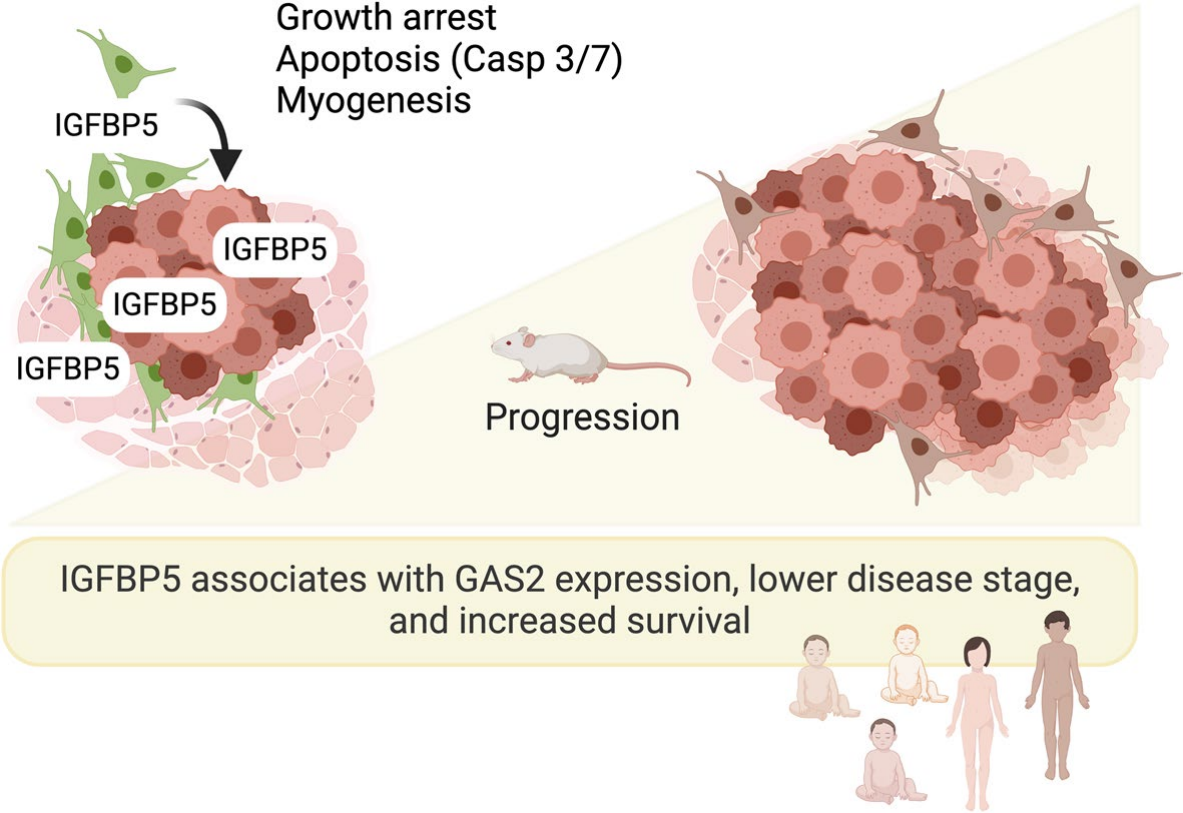

**Supplementary Information:**

The online version contains supplementary material available at 10.1186/s12964-025-02171-6.

## Background

Cancer cells reside in a tumor microenvironment with extracellular matrix (ECM), fibroblasts, vasculature, and immune cells. How these distinct components engage in the construction of local ecosystems controlling disease progression and therapy resistance in epithelial-derived cancers is outlined by multiple studies [1]. The corresponding mechanisms in malignancies of mesenchymal origin such as rhabdomyosarcoma (RMS) are less investigated.

RMSs belong to a group of small round blue cell tumors and are rare, heterogeneous childhood tumors of skeletal muscle lineage [2]. The main histotypes are historically referred to as embryonal and alveolar, but with molecular profiling it is nowadays more precise to distinguish fusion-negative RMS from fusion-positive, with the latter being diagnosed by detection of *PAX3/7-FOXO1* gene fusions. Such detection of pathognomonic fusion genes aids not only diagnostics, but is also informative from a prognostic perspective [3]. Still, how disease-promoting fusion genes and deregulated growth factor signaling unwire cellular differentiation is incompletely understood in RMS and other pediatric cancers [4, 5].

It was early suggested, though, that both *PAX3* fusions and wild-type *PAX* genes suppress apoptosis in RMS [6]. Apoptosis is one of many regulated cell death pathways and is partly controlled by executioner caspases like caspase 3, which in turn requires cleavage to become active [7, 8]. In a recent collaboration, we demonstrated that mechanical confinement and discoidin domain receptor 1 (DDR1) signaling regulate collagen-induced apoptosis in RMS cells [9]. Modeling in 3D coculture systems revealed that neighboring fibroblasts promoted proliferation and inhibited apoptosis of RMS cells, and we proposed a mechanism explaining why RMS grows in and metastasizes to low fibrillar collagen microenvironments.

These findings must however be further investigated in a context-specific manner where different stromal cell subsets coexist and coevolve over time. Patient age, sex and cell of origin are typical baseline characteristics that are likely to vary between mesenchymal tumors. RMS heterogeneity can also be exemplified by the rare occurrence of adult entities with distinct biology and treatment resistance mechanisms [10]. In addition, the anatomic location should be considered not only from a resection perspective, but also regarding density and reactivity of tumor-surrounding stroma [11]. Therefore, to what extent tumor-surrounding stroma regulates apoptosis, myogenic differentiation or other cellular processes involved in RMS initiation and progression remains to be determined.

Notably, it is well recognized that cancer-associated fibroblasts facilitate tumor progression in multiple human malignancies [12]. The corresponding cell types in sarcomas are less defined. ECM production and matricellular proteins are, however, known to be essential also in RMS where for example the matricellular protein connective tissue growth factor (CTGF) prevents apoptosis [13]. Regardless of the cellular source of stromal components, the bioavailability of matricellular proteins is tightly controlled by neighboring ECM, which is often produced by mesenchymal cells in a reactive state. These cells engage inflammatory cells, and growth factors like fibroblast growth factors (FGFs) and platelet-derived growth factors (PDGFs) are activated [14, 15].

Another deregulated growth factor signaling pathway of importance in RMS is the insulin-like growth factor (IGF) system [16–21]. Both IGF-II and IGF-IR are known to be overexpressed in RMS, and IGF-II was early found to be a critical player in stimulating cell proliferation and motility [22]. Hence, therapeutic targeting of IGF signaling in sarcomas has been thoroughly explored in multiple studies [23–26], and neutralization of IGF-II was recently suggested as an alternative to anti-IGF-IR therapies in RMS [27]. Unfortunately, the complexity of the IGF pathway is still hard to depict from a therapeutic perspective.

Healthy skeletal muscle cells depend on IGF signaling for their normal proliferation and differentiation [28], but there are also six binding proteins (IGFBP1-6) with either IGF-dependent or IGF-independent functions to consider [29–31]. IGFBP6, which binds with high affinity to IGF-II, can for example decrease RMS cell number [32, 33], and high levels of IGFBP5 are likely to neutralize IGF-I activity, which can lead to cell death [34, 35]. Moreover, fibroblast ECM is known to locally increase IGFBP5 levels and could thereby regulate IGF-1 bioavailability [36].

The role of IGFBP5 in differentiation and apoptosis was early studied in C2 myoblasts [37, 38]. IGFBP5 is dramatically induced during myogenesis, and exogenously added IGFBP5 stimulates myoblast differentiation in the presence of IGF-I [39]. Knockdown of *IGFBP5* is reported to impair myogenesis and it also suppresses IGF-II expression [40]. Other studies have suggested that *IGFBP5* is a target for microRNA-206, which may regulate differentiation of fusion-negative RMS [41]. *IGFBP5* is also part of the myogenic transcription program activated by the PAX3-FKHR fusion gene in alveolar RMS [42]. It has further been shown that *IGFBP5* is differentially expressed in two clones of the RD cell line with different myogenic differentiation potential [43].

The physiologically balanced expression levels of IGF receptors, ligands and binding proteins are often disrupted in disease. Our present study uses diverse sets of model systems and patient material to demonstrate that mesenchymal stromal cells nourish high IGFBP5 levels in the tumor microenvironment and promote myogenic identity of RMS cells. The data further supports that IGFBP5 provides anti-tumor growth signals involving growth arrest and apoptosis. To better define stromal cell subsets and their role as potential modulators of tumor growth in RMS could therefore be a new strategy in the development of more successful combination therapies.

## Methods

### Patient cohorts

Clinicopathological correlations were performed in three different patient cohorts based on gene expression analysis. Data from the RMS discovery cohort (Davicioni, u133a platform, *N* = 147) was downloaded from the R2 Genomics Analysis and Visualization Platform (https://hgserver1.amc.nl/cgi-bin/r2/main.cgi) and has been described before [44]. Similarly, the RMS validation cohort (ITCC/CIT: Innovative Therapies for Children and adolescents with Cancer/Carte d'Identit e des Tumeurs gene expression profile dataset, *N* = 101) has been previously described [3, 45], and was downloaded from BioStudies (https://www.ebi.ac.uk/biostudies/) using accession E-TABM-1202. The original array data was normalized by quantile normalization in the RMA function in R and non-varying probes were filtered out by nsFilter. Data from the Pan-Can STS cohort (*N* = 255) is publicly available, and the data was obtained and processed as previously described according to standard transcriptomics with R [46–48]. The UKCCSGRMS tissue microarray has been previously described [49].

### Analysis of *IGFBP5* levels in datasets

The HTCA database was used for visualizing *IGFBP5* expression levels in normal pediatric and adult tissue and single cells [50]. Obtained results were generated by the search term “IGFBP5” in the interactive atlas on https://www.htcatlas.org/genesearch.

*IGFBP5* expression data from mouse C2C12 cells with three biological replicates/group was obtained from the NCBI website/GEO Profiles selecting profile GDS2412/1452114_s_at (https://www.ncbi.nlm.nih.gov/geoprofiles/31864309).

For *IGFBP5* levels in RMS, the ITCC/CIT gene expression profile dataset containing 101 RMS samples profiled using the Affymetrix chip HG-133plus2 was used. 17 normal muscle samples profiled using the same Affymetrix chip were obtained from Gene Expression Omnibus (GEO): accession GSE7307 (GSM175882, -83, -84, GSM175940, GSM176310, -13, -17), GSE3526 (GSM80790, -91, -92), GSE2328 (from GSM42732, -33, -38), GSE5110 (GSM103559, GSM103560, GSM103563, GSM103565). We also obtained 29 expression profiles from mesenchymal stem cells (MSC): extracted from bone marrow GSE9593 (GSM242185, GSM242650, -51, -52, -53, -66, -67, -68, -69, -72,-73, -74, -75), GSE26272 (GSM645185, -86, -89, -90), or umbilical cord blood GSE13491 (from GSM340079 to GSM340090). In addition, 63 fibroblast samples were included (GSE63626). Analysis was done using the R software version 4.2.1 (www.r-project.org) and the Bioconductor 3.15 packages. Annotation data for HG-133plus2 was obtained from the package hgu133plus2.db version 3.13. Data were log2-transformed following import. Normalization across different datasets was performed using the normalizeBetweenArrays function from the limma package. To account for potential batch effects, batch correction was applied using the removeBatchEffect function from ComBat. Principal component analysis (PCA) and boxplot visualizations, incorporating both all genes and eight housekeeping genes (GAPDH, ACTB, HPRT1, B2M, RPL13A, TBP, PPIA, RPL19) were used to demonstrate that the data were comparable across datasets (Additional file S1). The probe with highest value was selected if multiple probes were mapped to *IGFBP5*. The Wilcoxon rank-sum test was used to compare gene expression levels between RMS and other cell types by the function geom_signif using the package ggsignif version 0.6.4.

For clinicopathological analysis, *IGFBP5* expression levels were analyzed regarding tumor stage, fusion gene status and survival in the discovery cohort and in the validation cohort. The Student´s *t* test was used for statistical comparisons. Survival analysis was carried out by the Kaplan–Meier method with the groups tested divided by the median of gene expression, and the log-rank test was used for comparison between groups.

The discovery and validation cohort samples were divided into three groups based on *IGFBP5* expression, and the lower and upper group were used for DEG analysis done by DESeq2(v1.40.2) in R. Genes with FC higher than 1.5 and lower than -1.5 and *P* < 0.05 were kept. The *P* value was adjusted by the BH method.

### Single cell RNAseq analysis

Processed dataset and annotation tables were obtained from GEO under accession GSE195709. Details on how these datasets were processed can be found in the methods section of the corresponding paper (PMID: 35982179). Function VlnPlot and DotPlot of Seurat(v.4.0) were used to draw the corresponding plots.

### Cell culture

Cells were kept at 37 °C in a humidified 5% CO_2_ atmosphere. High glucose Dulbecco's Modified Eagle Medium (DMEM) supplemented with 10% FBS, 2 mmol/L glutamine, 100 U/mL penicillin, and 100 µg/mL streptomycin was used for all cell lines unless otherwise stated. RD cells were obtained from ATCC (CCL-136) at low passage. Other RMS cells have been presented elsewhere [11]. Mycoplasma testing and cell line authentication for RD, RH36 and Ruch2 cell lines were performed through Eurofins genomics. For primary cell isolations of mesenchymal stromal cells with very limited life span, these were instead confirmed negative for mycoplasma by Hoechst DNA stain visualized by immunofluorescence microscopy. Adult and fetal human dermal fibroblasts (HDF, 106-05A, 106-05F) were purchased from Merck (Cell Applications inc.) and amplified in ready to use Fibroblast growth medium (116–500, Merck) before freezing. For experimental use, all cells were adapted to DMEM with supplements as above.

### Xenograft studies in mice

Experimental procedures were approved by the local committee for animal experiments (N220/14). 1.5 million RD cells in PBS were implanted subcutaneously into the flank of 8-week-old female SCID mice. Tumors were monitored based on tumor size (width^2^xlengthx0.52). Orthotopic injections into the hindlimb were similarly performed after isoflurane anesthetization. For the latter injections, buprenorfin (Temgesic®) was given subcutaneously as pain relief at a dose of 0.05 mg/kg before injection and thereafter at recommended dose interval the first 24 h. All experiments were performed in accordance with the approved protocol and other relevant guidelines and regulations, and terminated before the growing tumors reached a maximum size of 1 cm^3^ or hampered natural behavior, such as spontaneous climbing in the cage. Statistical analysis was done by Student´s *t* test (two groups) or one-way ANOVA (three groups) where *P* < 0.05 was considered statistically significant.

### Isolation and characterization of primary stromal cells

Mouse stromal cells were isolated from hindlimb skeletal muscle and lung of adult SCID mice. The dissected tissues of interest were collected under aseptic conditions, placed in serum-free DMEM, and then cut into small pieces with scalpels. Digestion medium with 1 × collagenase + hyaluronidase (Stem Cell Technologies) and 1 × dispase (Stem Cell Technologies) was prepared just before use in serum-free DMEM and 10 mg/ml DNase 1. The small tissue pieces were incubated in digestion medium at 37 °C for 3 h with occasional shaking. Thereafter, the dissociated tissue was passed through an 18G needle attached to a 5 ml syringe (3 × in and out). The procedure was repeated with a 20G needle and a 23G needle to obtain a single cell suspension. Cells were then diluted in PBS and passed stepwise through 100 µM, 70 µM and 40 µM cell strainers before pelleting and subsequent transfer to T25 cell culture flasks with 10%FBS/DMEM. After cell culture expansion to T75 flasks, fluorescence-activated cell sorting for mouse pdgfrβ expression was performed: Cells to be sorted were trypsinized, resuspended in PBS, passed through a 40 µm cell strainer and counted. After centrifugation 5 min at 1000 rpm, they were resuspended in staining buffer (PBS/0.5% BSA) using 100 µl to 1 million cells. PE-labelled anti-mouse CD140b (1:20, Affymetrix eBioscience) was added as primary antibody in parallel to a PE-labelled IgG control antibody (1:20, Affymetrix eBioscience). Following 30 min incubation in the dark at 4 °C, 2 × washing in sorting buffer (PBS, 2 mM EDTA, 1% FBS, 25 mM HEPES) was carried out before sorting for PE + cells. Cultures were confirmed to be more than 99% positive for pdgfrß from normal hindlimb muscle. For cultures of tumor-associated stromal cells, two subsequent sortings for mouse pdgfrβ were performed within approximately two weeks (every time when cultures were 95% confluent). As soon as there were enough cells (3–4 passages), they were used for preparing the conditioned medium.

### Immunostaining

Immunohistochemistry of human RMS tissue microarray with 0.6 mm diameter cores was according to the manufacturer´s protocol for cleaved caspase 3 (9661S, Cell signaling).

Characterization of isolated stromal cells from normal mouse hindlimb and/or RD xenografts implanted in mouse hindlimb was performed by immunostaining of early passage cells for desmin (M0760, clone D33, 1:200, DAKO). Subconfluent cells on cover slips in 6 well plates were washed in PBS, fixated in 4% PFA for 15 min, washed in PBS, permeabilized with 0.1% Triton-X for 30 min, blocked with ready-to-use protein block (DAKO) and incubated with primary antibody for 30 min at room temperature. Following a PBS wash, a secondary Alexa 594 antibody (Life Technologies) was applied for 30 min at room temperature followed by washing in PBS and water before mounting with Prolong gold antifade mountant with DAPI (ThermoFisher Scientific). The AxioVision Rel. 4.6 Software (Carl Zeiss) was used for automated quantifications of immunostaining.

Cryopreserved orthotopic hindlimb xenografts were sectioned and fixated in ice-cold acetone for 10 min followed by incubation in ready-to-use protein block (DAKO). RMS tumor cells were detected by desmin antibodies (M0760, clone D33, 1:100, DAKO), blood vessels with podocalyxin antibodies [11], and mesenchymal cells with pdgfrß antibodies (3169, 1:50, Cell Signaling Technology). Species-matched secondary ALEXA antibodies were from Molecular Probes.

### CyQuant cell proliferation assay

Cells were seeded in 10% FBS/DMEM at sub-confluency (1000 cells/well) in 96 well plates and 4 replicates/condition. 24 h later, treatment was initiated. Plates with cells were then washed in PBS and placed in -80 °C for at least 24 h, and thereafter analyzed using the CyQuant proliferation assay (Life Technologies Corp) according to the manufacturer’s instructions. Cell proliferation was analyzed using a Spark 10 M Multimode Microplate reader (Tecan). The amount of nucleic acid present in lysed cells was normalized to the amount present in a parallel plate frozen at time 0 h when treatment was initiated. Similarly, for IGFBP5 treatment, cells were seeded in a 96-well plate (3500 cells/well) and treated with 5 μg/ml of IGFBP5 (875-B5, Bio-Techne Ltd) in 10% FBS/DMEM for 24 h. Cell proliferation was then measured as above. All CyQuant cell proliferation experiments were analyzed using the Student’s *t* test where *P* < 0.05 was considered to be statistically significant. Data is presented as the mean ± SD.

### Myogenic differentiation analysis

Gene expression levels of MYF5 and MYF6 in RD cells and RH36 cells were explored in the Cancer Cell Line Encyclopedia (CCLE) on the Cancer Dependency Portal (DepMap) https://depmap.org/portal/ccle/. For experimental differentiation studies, cells were seeded in 10% FBS/DMEM at sub-confluency in two 6-well plates. The following day, the cell medium in both plates was replaced by DMEM containing 2% horse serum. After 6 h, cells in the first plate were washed in PBS and lysed (Time 0, baseline control). After 72 h, cells in the second plate were washed with PBS, lysed, and harvested for RNA extraction.

### RNA extraction, cDNA synthesis and qPCR

RNA was extracted from 6 well plates using the GeneElute™ Mammalian Total RNA Miniprep Kit (Sigma-Aldrich) protocol including an on-column DNase digestion step. For cDNA synthesis, SuperScript II First-Strand Synthesis System for RT-PCR (Invitrogen) was used. SYBRgreen Universal PCR Master Mix (Applied Biosystems) was used in the PCR reaction with the following Qiagen QuantiTect Primers: *GAPDH* (Hs_GAPDH_1_SG, Cat.NO.QT00079247) and *IGFBP5* (Hs_IGFBP5_1_SG, Cat.NO.QT00047530). *MYF6.*

was detected with primers from Sigma-Aldrich (Forward 5’-AGAAAATCTTGAGGGTGCGG, Reverse 5’-CCCCTGGAATGATCGGAAAC). The thermal protocol started with DNA polymerase activation at 95 °C for 10 min, followed by 40 melt-anneal-extend cycles of 95 °C for 15 s and 60 °C for 1 min. Gene expression was analysed by the 2^-(ΔΔCt) method using the cycle threshold (Cq) values from the Bio-Rad CFX Manager software program and *GAPDH* as reference gene. Gene expression was statistically analyzed using GraphPad Prism. A ratio paired T-test was used where *P* < 0.05 was the threshold for significance. Gene expression is presented as fold gene expression 2^-(∆∆Ct) with mean ± SEM (normalized to *GAPDH*).

### Conditioned medium exposure and gene expression analysis with the Clariom D platform

Mouse experiments were preceded with a conditioned media exposure step as indicated, and paralleled with gene expression analysis of RD cells. All conditioned media exposure experiments were performed using normal growth medium in cell culture. The cell supernatants were collected 24 h after changing to fresh media in subconfluent cultures, irrespective of the cell type used, without splitting the cells. A brief centrifugation was performed to make sure that no floating cells and debris were passed along. One set of parallel flasks of primary skeletal muscle stromal cells (p.SkMSc) from mouse were in turn pre-exposed to RD cell conditioned medium for 24 h before the other medium replacement to fresh cell culture medium. These latter cells were referred to as pre-stimulated primary skeletal muscle stromal cells (prestim p.SkMSc). Conditioned medium from these primary cells were then subsequently collected and applied on RD cells in culture for comparative analysis with that from conditioned medium collected from tumor-associated primary skeletal muscle stromal cells isolated from hindlimb muscle. Normal growth medium kept in an empty cell culture flask for the same amount of time in the incubator was used as negative control.

Gene expression changes in RD cells after overnight exposure (16 h) to conditioned medium as described (normal growth medium, cell supernatant) from mouse skeletal muscle stromal cells, compared to control medium, were analyzed by high resolution microarray-based Clariom D gene expression analysis. Total RNA (4 replicates/condition) was quality checked on Agilent Technologies 2200 Tapestation and RNA quantity was measured using NanoDrop ND-1000 Spectrophotometer (Thermo Fisher Scientific). Biotinylated DNA targets were prepared from 150 ng total RNA using the GeneChip WT Plus Reagent Kit (Affymetrix, Thermo Fisher Scientific) according to the manufacturer's instructions. Hybridization, washing and staining was carried out on Affymetrix GeneChip Clariom D human arrays (Affymetrix, Thermo Fisher Scientific), using Affymetrix GeneChip® Fluidics Station 450, according to the manufacturer’s protocol. The fluorescent intensities were determined with Affymetrix GeneChip Scanner 3000 7G.

Data analysis was performed using Expression Console Software (v1.4.1). Raw data was background corrected, normalized, summarized and log2 transformed using the Robust Multi-array Average (RMA) algorithm. Unpaired 2-sided Student's *t* tests were used for comparisons between control and treatment group to identify differentially expressed genes. To address multiple testing, q values were included (calculated by the qvalue function from the qvalue Bioconductor package). The data is available under the GEO accession number GSE283825.

### Caspase 3 activity assay

Caspase 3 activity of cells was measured by a caspase 3 colorimetric activity assay kit (Merck Millipore, APT165). 20,000 cells were seeded in 12-well plates and the treatment with 5 μg/ml IGFBP5 started when cells reached 80% confluence. After 24 h, all cells were lysed with 90 µl 1X lysis buffer for 10 min on ice. Debris was removed by centrifugation at 10,000 rpm for clear supernatant, of which 70 µl was mixed with 20 µl 5X assay buffer and 10 µl caspase 3 DEVD-*p*NA substrate. 5X assay buffer diluted in water was used as blank control. The assay mixture was incubated in a 96-well plate at 37 °C for one hour. Thereafter, the samples were read at 405 nm in a microplate reader (Varioskan). Caspase 3 activity was calculated based on the *p*NA standard curve and determined by normalization to the total protein concentration of each sample as measured with a Bradford protein assay kit (Thermo Scientific, 23,200, Stockholm, Sweden). A Student´s *t* test was used where *P* < 0.05 was the threshold for significance. Results are presented as mean *p*NA released from DEVD-*p*NA (µmol/mg) relative to protein concentration ± SEM.

### Construction of IGFBP5 overexpressing and knockdown cell lines

To construct IGFBP5 overexpressing cell lines, hygromycin and GFP-labelled mammalian gene expression lentiviral vectors with the human *IGFBP5* coding region sequence or stuffer (as control) were purchased from VectorBuilder GmbH. The *IGFBP5* or stuffer vector was co-transfected (Lipofectamine 2000 Transfection Reagent, Invitrogen) with psPAX2 and VSV-G vectors (Addgene) into HEK293T cells (ATCC). psPAX2 was a gift from Didier Trono (Addgene plasmid # 12,260; http://n2t.net/addgene:12260; RRID:Addgene_12260), whereas pCMV-VSV-G was a gift from Bob Weinberg (Addgene plasmid # 8454; http://n2t.net/addgene:8454; RRID:Addgene_8454) [51]. At 48 h post transfection, the virus-containing media from HEK293T cells were applied to the different target cells (RD and RH36). After 24 h exposure, the media on target cells were changed to growth medium containing 1 μg/ml hygromycin B (Thermo Scientific, J67371.8EQ, Stockholm, Sweden) for selection. The selection lasted for 10 days with media change every second day. Thereafter, the transduced target cells were cultured in medium free of hygromycin B.

### Cell cycle analysis

Cells were seeded and treated at sub-confluency in 6-well plates. Before analysis, attached cells were trypsinized and pooled with floating cells. Pelleted cells were resuspended and washed with 2 ml PBS after centrifugation at 1000 rpm for five minutes. Following another centrifugation step, the cells were resuspended in 4% PFA for fixation overnight at + 4 °C. After centrifugation at 3000 rpm for five minutes, and washing with PBS, the fixed cells were subjected to permeabilization in 150 µl 0.1% Triton X-100. Permeabilized cells were incubated with 1 µl of 1 mg/ml DAPI for 105 min in the dark before cell cycle analysis with NovoCyte 3000 (Agilent Technologies).

### Analysis of IGF1 pathway

For IGF1 pathway analysis, the discovery cohort was divided into three groups, whereas the validation cohort was divided into four groups based on IGFBP5 expression, and the lower and upper group were used for the analysis of gene in IGF1 pathway. The gene set of IGF1 pathway was collected from GSEA (BIOCARTA_IGF1_PATHWAY, https://www.gsea-msigdb.org/gsea/msigdb/human/geneset/BIOCARTA_IGF1_PATHWAY.html) and results visualization was done by ggplot2(v3.4.4) in R. The Student´s t test was used for statistical comparisons. The *P* value was adjusted by the BH method.

## Results

### Tumor growth-suppressive effects of orthotopic stromal cells are lost after in vitro or in vivo exposure to RMS cells.

**Fig. 1:**
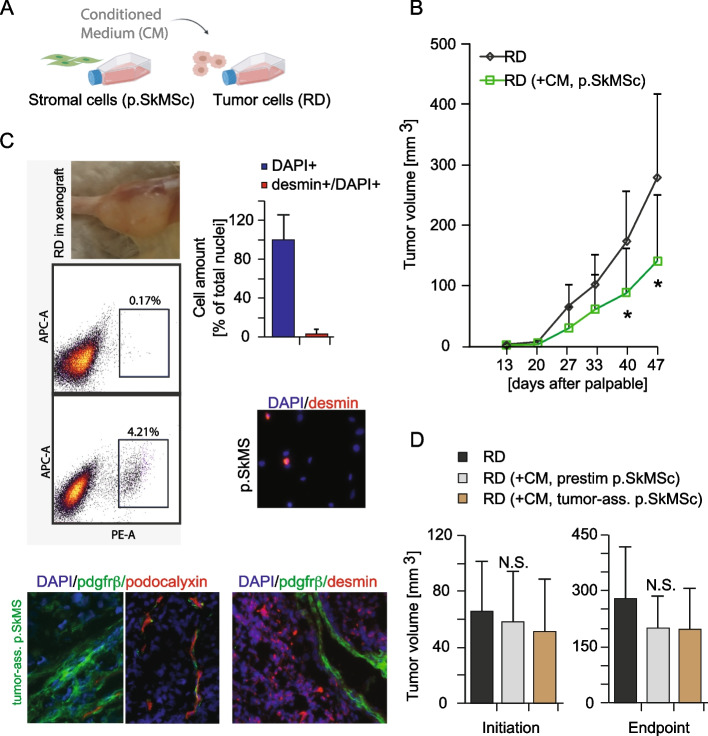
Tumor growth-suppressive effects of orthotopic stromal cells are lost after in vitro or in vivo exposure to RMS cells. **A**. Schematic overview of the experimental design. The sarcoma cell line RD was exposed to control medium or conditioned medium (CM) from freshly isolated naïve primary skeletal muscle stromal cells (p.SkMSc) prior subcutaneous injection into the flank of SCID mice. **B**. Tumor growth of RD xenografts with or without prior exposure to CM from p.SkMSc. N ≥ 9/experimental group. **C**. Tumor-associated p.SkMSc were isolated from orthotopic xenografts by enzymatic digestion, expansion in cell culture and sorting by FACS for mouse pdgfrβ expression. Characterization of xenografts demonstrated pdgfrβ + stromal cells, podocalyxin + endothelial cells and desmin + muscle and tumor cells. The vast majority of the isolated cells was negative for desmin. **D**. Tumor growth of subcutaneous RD xenografts after exposure to CM from in vitro*-* (prestim) or in vivo*-*educated tumor-associated (tumor-ass.) orthotopic stromal cells. N ≥ 9/experimental group. *P < 0.05; Student´s *t* test (two groups). N.S. not significant; one-way ANOVA (three groups). Data is presented as the mean ± SD

To molecularly examine mesenchymal stromal cell-mediated effects on RMS cells, a mouse model system was used where mouse stromal cells of the same skeletal muscle origin were differentially exposed to RMS cells. The education was performed either in vitro by conditioned medium or orthotopically in vivo during tumor development in hindlimb skeletal muscle. In the first setup, conditioned medium from naïve stromal cells was applied on RD cells (Fig. 1A) the day before subcutaneous mono-injection. The RD cells were exposed to the conditioned medium for 16 h and the medium was washed away during the cell preparation step just before tumor cell injection. Tumor growth was then monitored, and the resulting tumors were consistently smaller compared to control (Fig. 1B).

Next, tumor-associated stromal cells were isolated by enzymatic digestion of RD tumors grown in mouse hindlimb. Adherent cells were expanded in cell culture and separated from malignant cells by fluorescence-activated cell sorting specific for mouse pdgfrβ (Fig. 1C). Among the pdgfrβ + cells, the majority was negative for desmin, which indicated a more fibroblast/pericyte-like than myoblast-like phenotype. In contrast to the results from the first experimental setup with naïve stromal cells, no stromal cell-mediated suppressive effects on tumor growth were detected following exposure to conditioned medium from tumor-associated stromal cells before RD cell injection, and similar results were obtained using conditioned medium from short-term in vitro pre-stimulated skeletal muscle stromal cells (Fig. 1D). Tumor growth-suppressive effects were also not detected when orthotopic skeletal muscle stromal cells were co-injected with RD cells (Additional file S2). Accordingly, stromal-derived growth-suppressive effects on RMS growth were shown to be transient and easily lost in the presence of neighboring malignant cells.

### TGFβ–inducible genes are downregulated in RMS cells after exposure to tumor growth-suppressive mesenchymal stromal cells.

A Clariom D platform-based gene expression analysis of RD cells exposed to conditioned medium from tumor growth-suppressive mesenchymal stromal cells identified several significantly deregulated genes. The TGFβ–inducible genes *CCN2*/Connective tissue growth factor (*CTGF*) and *SERPINE1*/plasminogen activator inhibitor 1 (*PAI-1*) were both found among the top 10 downregulated genes (Additional file, Table 1). These genes have multiple established functions in tumorigenesis across tumor types, and in publicly available soft tissue sarcoma data sets, they were shown to associate with decreased survival (Additional file S3A).

Beyond regulatory activities on differentiation, migration, angiogenesis and immunosuppression, CTGF has particularly been shown to control RMS tumor cell survival [13]. This finding was confirmed in a cell proliferation assay where an increased RMS cell survival was observed in the presence of CTGF after three days in culture under starvation conditions (Additional file S3B). Moreover, when deregulated genes in high versus low *CCN2*-expressing tumors in two independent RMS cohorts were identified, one of the top candidate genes was *SERPINE1* (Additional file S3C-D), which suggested co-regulatory expression of the TGFβ–inducible genes *CTGF* and *SERPINE1* in RMS.

### *IGFBP5* is induced by tumor growth-suppressive mesenchymal stromal cells and dampens RMS cell proliferation.

**Fig. 2:**
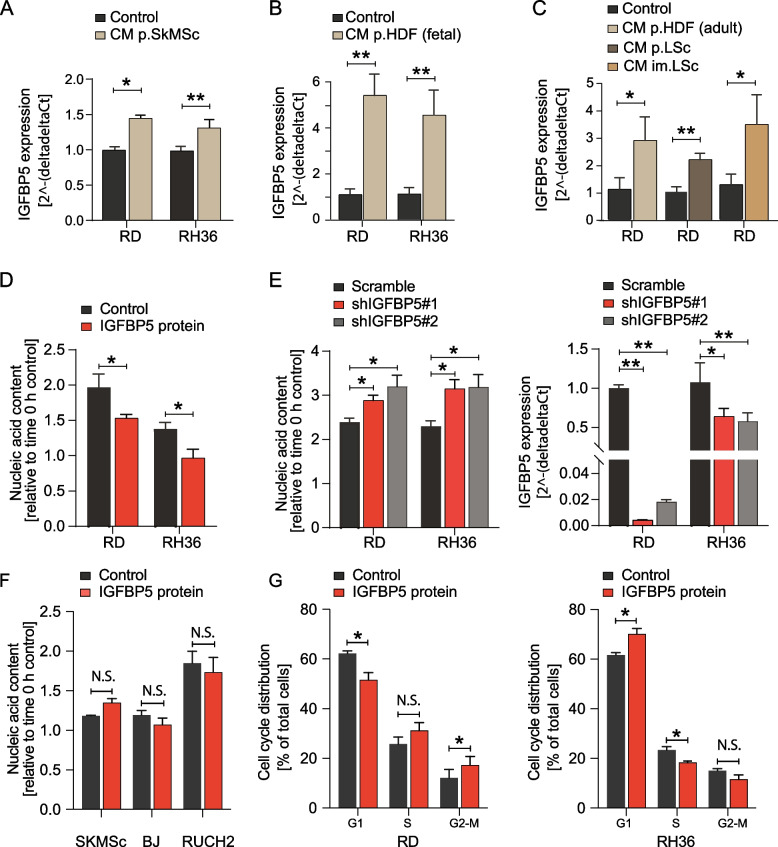
IGFBP5 is induced by tumor growth-suppressive mesenchymal stromal cells and dampens RMS cell proliferation. **A ***IGFBP5* gene expression analysis by qPCR in RD and RH36 cells after exposure to CM from primary skeletal muscle stromal cells. **B ***IGFBP5* gene expression analysis by qPCR in RD and RH36 cells after exposure to CM from primary HDF stromal cells of fetal origin. **C ***IGFBP5* gene expression analysis by qPCR in RD cells after exposure to CM from primary HDF stromal cells (adult origin) and stromal cell isolations from mouse lung (primary cells and spontaneously immortalized). **D **RD and RH36 cell proliferation after 5 μg/ml IGFBP5 protein treatment for 24 h in regular growth medium. **E **Cell proliferation and *IGFBP5* gene expression after knockdown for IGFBP5 in RD and RH36 cells. **F **SkMSc, BJ and RUCH2 cell proliferation after IGFBP5 protein treatment for 24 h in regular growth medium. **G **Cell cycle analysis in RD and RH36 cells treated with IGFBP5 protein. *P < 0.05, **P < 0.001, N.S. not significant. Data is presented as the mean ± SD if not otherwise stated (see Methods section for statistical methods)

From the Clariom D-based gene expression analysis, *IGFBP5* was identified as the major upregulated gene in RMS cells exposed to tumor growth-suppressive stromal cells (Additional file, Table 1). The induced *IGFBP5* expression was also confirmed by qPCR, and in settings where additional stromal cell isolations of various origin (primary skeletal muscle stromal cells, primary dermal fibroblasts of fetal and adult origin, primary lung fibroblasts, immortalized lung fibroblasts) and RMS cell lines were included (Fig. 2A-C). Treatment of RD and RH36 cells with 5 μg/ml IGFBP5 protein also decreased cell proliferation (Fig. 2D), whereas knockdown of *IGFBP5* increased cell proliferation (Fig. 2E). No response on total nucleic acid content to IGFBP5 treatment was detected in primary stromal cells, neither skeletal muscle stromal cells nor BJ fibroblasts, or in the botroid RMS cell line RUCH2 (Fig. 2F). Complementing experiments showed an altered cell cycle distribution in response to IGFBP5 protein treatment both for RD cells and RH36, with RD cells arresting in G2-M, and RH36 cells arresting in G1 (Fig. 2G). Whether these observed responses on cell proliferation and/or survival could be translated to the in vivo setting was next to be investigated in patient material.

### *IGFBP5* associates with the caspase 3 substrate *GAS2* in patient material and induces caspase 3/7 activity.

**Fig. 3:**
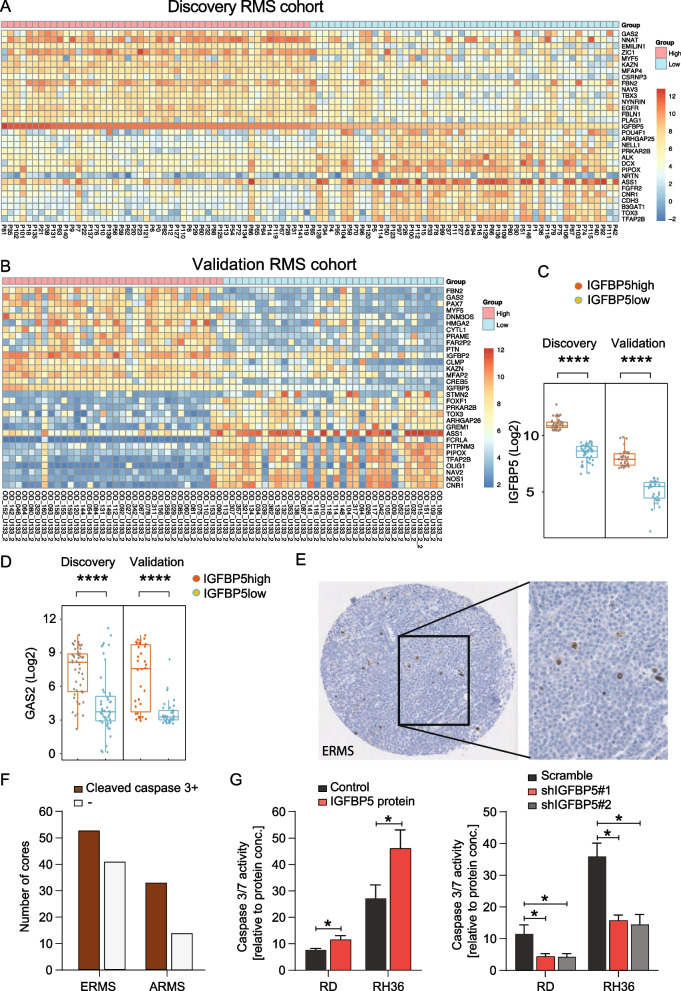
IGFBP5 associates with the caspase 3 substrate GAS2 in patients and induces caspase 3/7 activity. **A**, **B **Heatmap of differentially expressed genes in high versus low *IGFBP5*-expressing tumors in the discovery cohort and the validation cohort. High versus low gene expression was defined using upper and lower tertile (**C**). **D ***GAS2* expression in the discovery and validation cohort comparing highest and lowest tertile of *IGFBP5* expression. **E **Immunostaining (IHC) for cleaved caspase 3 in human RMS tissue cores. **F **Number of RMS tissue microarray cores (2 per case) with positive or negative IHC staining for cleaved caspase 3. **G**. Caspase 3/7 activity in RD and RH36 cells after 5 μg/ml IGFBP5 treatment (left) and IGFBP5 knockdown (right). ****P < 0.0001, *P < 0.05. Data is presented as the mean ± SD if not otherwise stated (see Methods section for statistical methods)

Differentially expressed genes that characterize *IGFBP5*-expressing tumors were next visualized in heatmaps with data from two independent cohorts (Fig. 3A-C). The caspase 3/7 substrate *GAS2* was hereby recognized as the most differentially expressed gene across the cohorts (Fig. 3D). *GAS2* is reported to increase susceptibility to apoptosis and regulate the associated cell shape changes [52]. Therefore, potential links between *IGFBP5* and apoptosis were investigated. Gene expression analysis of caspase 3 in both the discovery cohort and the validation cohort suggested more abundant levels in translocation-negative RMS (Additional file, Table 2). However, in tissue cores stained for the executing death protease cleaved caspase 3, the majority of both embryonal and alveolar cores was confirmed to be positive on the level of active protein. This indicated that apoptosis mediated by activated caspase 3 is likely to occur in both subtypes (Fig. 3E-F, Additional file S4).

Next, RMS cells were treated with 5 μg/ml IGFBP5 protein in cell culture, and caspase 3/7 activity was measured by ELISA. A significant increase in apoptosis was demonstrated both in RD and RH36 cells (Fig. 3G left), which was in line with the decreased cell proliferation/survival previously observed. In contrast, lentiviral sh*IGFBP5* knockdown in both RD and RH36 cells showed decreased caspase 3/7 activity (Fig. 3G right). Altogether, the data suggested that apoptosis mediated by activated caspase 3 is influenced by the presence of IGFBP5 in RMS.

### IGFBP5 supports myogenic identity

**Fig. 4:**
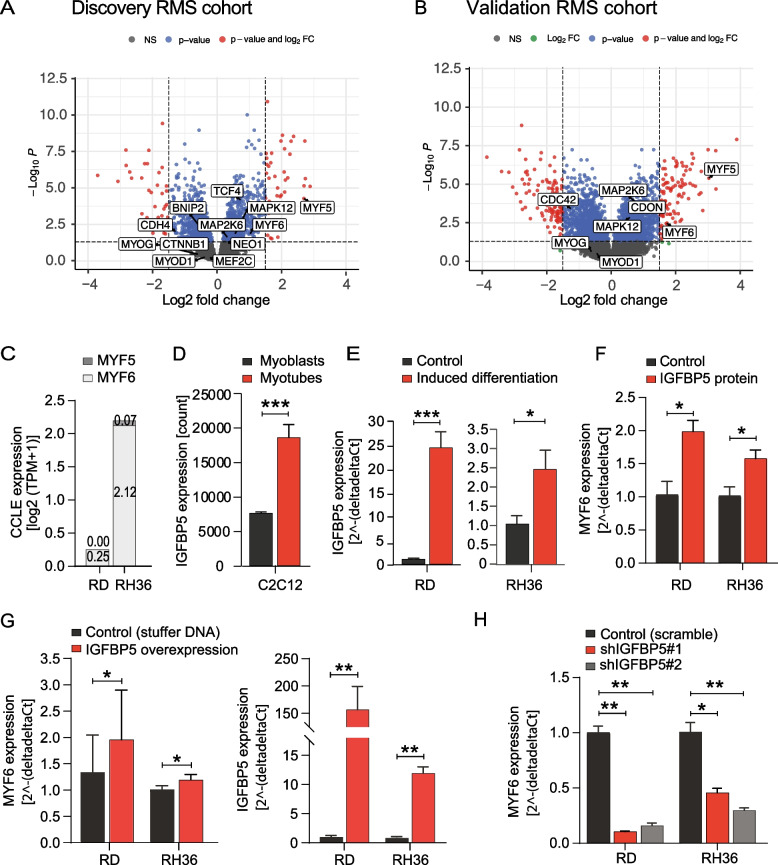
IGFBP5 supports myogenic identity. **A**, **B **Volcano plot of genes identified to be differentially expressed in cases with high versus low *IGFBP5* gene expression in the discovery and validation RMS cohorts. Differentially expressed genes are displayed with red dots (absolute value of Log_FC higher than 1.5 and the q-value lower than 0.05) and myogenic genes are indicated with names if at least one threshold was passed. High versus low *IGFBP5* gene expression was defined using cohort tertiles. **C **Gene expression levels of *MYF5* and *MYF6* in RD cells and RH36 cells (Cancer Cell Line Encyclopedia, DepMap). **D **Upregulation of *IGFBP5* during differentiation of normal myoblasts in vitro. **E **Upregulation of *IGFBP5* in RD and RH36 cells during induced myogenic differentiation in the presence of 2% horse serum for 72 h (RD) or one week (RH36). **F ***MYF6* expression in response to IGFBP5 protein treatment under normal growth conditions in RD and RH36 cells. **G ***MYF6* expression after lentiviral IGFBP5 overexpression in RD and RH36 cells. *IGFBP5* expression levels after selection were determined by qPCR. **H ***MYF6* expression after lentiviral shIGFBP5 knockdown in RD and RH36 cells. *P < 0.05, **P < 0.01, ***P < 0.001. Data is presented as the mean ± SD if not otherwise stated (see Methods section for statistical methods)

When RMS cohorts were analyzed for differentially expressed genes in *IGFBP5* high- versus low-expressing tumors, both *MYF5* and *MYF6* were significantly associated with high *IGFBP5* gene expression (Fig. 4A, B). A more detailed analysis of the IGF signaling pathway, with the same selected set of genes in both cohorts, further suggested a particular role for the docking protein IRS1 (Additional file S5). IRS1, or insulin receptor substrate 1, regulates skeletal myogenesis, and myoblasts with higher IRS1 levels have for example been reported to be eliminated during differentiation due to abnormal sustainment of IGF-1R activation [53]. Accordingly, the decreased proliferative and/or survival response to IGFBP5 in culture could presumably also be explained by increased myogenic differentiation.

To functionally explore myogenesis, a first screen of known myogenic differentiation factors expressed in the RD and RH36 cell lines was performed. In this analysis, *MYF6* was identified as the best candidate gene given its function in rather late myogenic differentiation and its detectable expression levels in both cell lines (Fig. 4C). IGF signaling and IGFBP5 have earlier been associated with myogenesis, and as indicated from the established C2C12 model system, it was evident that *IGFBP5* expression was strongly induced in myoblasts undergoing differentiation (Fig. 4D). *IGFBP5* was also significantly upregulated in both RD cells and RH36 cells when myogenic differentiation was experimentally induced (Fig. 4E). Analysis of *MYF6* expression levels in RMS cell lines after treatment with recombinant IGFBP5 protein further supported an increased myogenic identity (Fig. 4F). Finally, stable *IGFBP5* overexpression by lentivirus increased *MYF6* levels in RMS cell lines (Fig. 4G), whereas lentiviral sh*IGFBP5* knockdown decreased *MYF6* expression (Fig. 4H). Altogether, these results indicated that IGFBP5 supports the myogenic identity of cells both during normal developmental myogenesis and in RMS.

### Single cell RNA sequencing reveals distinct expression of *IGFBP5* across mesenchymal cell states in the RMS tumor microenvironment

**Fig. 5:**
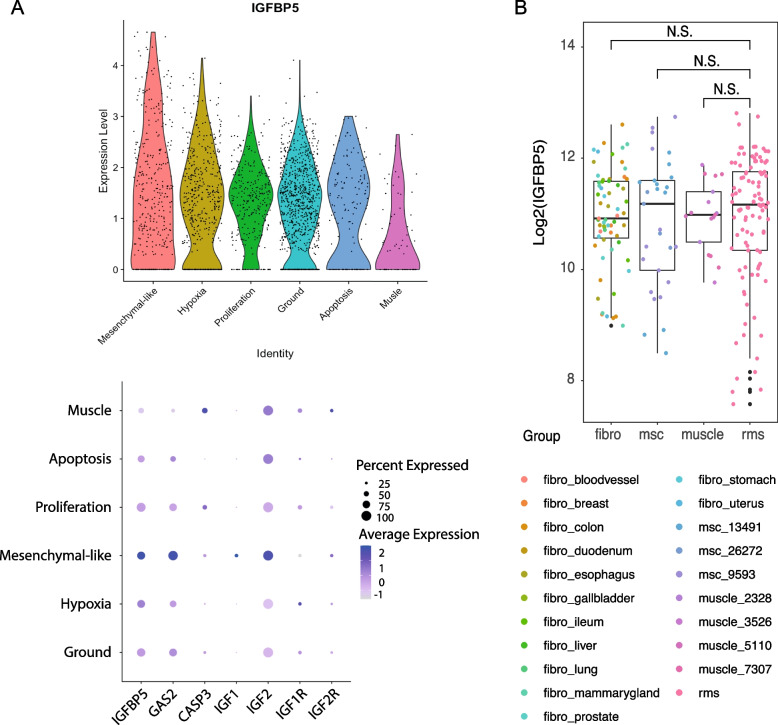
Single cell RNA sequencing reveals prominent IGFBP5 expression in mesenchymal-like cell states in the RMS tumor microenvironment. **A** Representative patient sample with expression of *IGFBP5* in single cells where cell identity for muscle, apoptosis, proliferation, mesenchymal-like and hypoxia is defined. Investigated genes of interest with a potential link to *IGFBP5* are displayed below and gene expression levels are visualized as percent expressed (size of dots) and average expression (color intensity). **B ***IGFBP5* expression levels in fibroblasts of different origin, mesenchymal stem cells, muscle and RMS. N.S. not significant; Wilcoxon rank-sum test

Single cell RNA sequencing data was next explored to characterize the expression pattern of *IGFBP5* across different cell types in the human RMS tumor microenvironment. Four cases with malignant cells that could be categorized into all phenotypes of proliferating, apoptotic, mesenchymal-like, and skeletal muscle cells, were identified for explorative analysis. The results revealed that *IGFBP5* expression was most prominent in cells defined as mesenchymal-like, in contrast to cells in a proliferative cell state (Fig. 5A, Additional file S6). Whether the sequencing data from these four patients is representative for the majority of RMS patients remains to be shown, but it supports a role for IGFBP5 in dampened RMS cell proliferation and/or cell cycle arrest.

*IGFBP5* expression levels in RMS cells, mesenchymal stem cells, and skeletal muscle, were next compared with data from cells defined as fibroblasts. Notably, *IGFBP5* levels in fibroblasts were highly diverse across samples and anatomic sites (Fig. 5B), and it is tempting to speculate that such diverse cells may provide different effects on tumor growth. Complementing analysis of data in the HTCA database demonstrated higher expression of *IGFBP5* in normal adult muscle versus fetal (Additional file S7A). Similarly, *IGFBP5* expression was higher in adult fast skeletal muscle cells versus fetal (Additional file S7B). The data thereby suggested a diverse expression of *IGFBP5* across different mesenchymal cell types, and consistently higher expression in adult cells versus fetal of the corresponding cell types.

### *IGFBP5* associates with favorable prognosis in pediatric RMS, but not in adult soft-tissue sarcoma

**Fig. 6:**
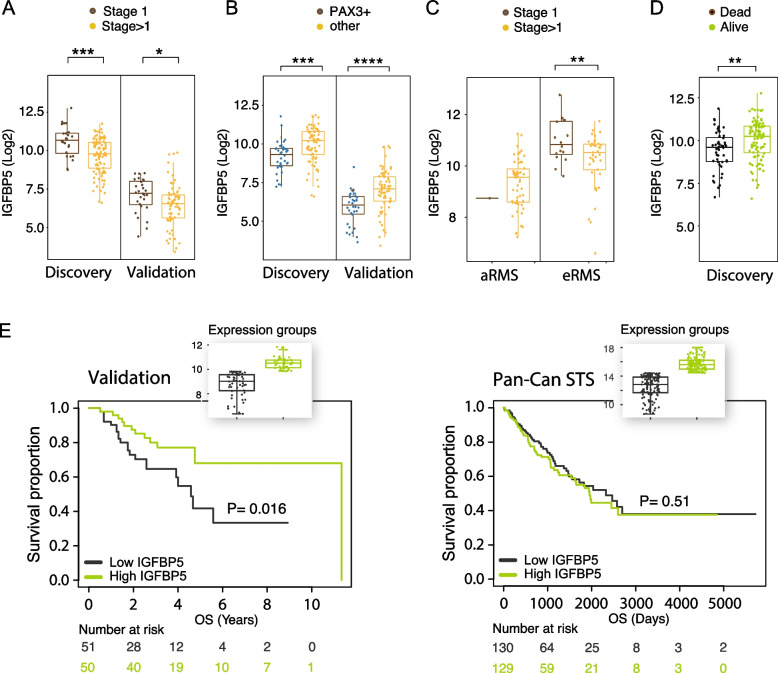
IGFBP5 associates with favorable prognosis in pediatric RMS, but not in adult soft-tissue sarcoma.** A ***IGFBP5* expression in the discovery RMS cohort (left) and the validation cohort (right) analysing patient stage (1 versus > 1) for all patients with stage information available. **B ***IGFBP5* expression in the discovery RMS cohort (left) and the validation cohort (right) analysing PAX3 fusion status (positive versus other) for all patients with fusion status available. **C ***IGFBP5* expression in the discovery RMS cohort analysing patient stage (1 versus > 1) for patients with the alveolar subtype (left) and the embryonal subtype (right). **D ***IGFBP5* expression in the discovery RMS cohort analysing patients alive versus not alive. **E** Kaplan-Meier survival analysis with log-rank test for *IGFBP5* expression in RMS (left) and adult soft-tissue sarcoma (right) with the latter cohort dominated by leiomyosarcoma, dedifferentiated liposarcoma, and undifferentiated pleomorphic sarcoma. Data presented with *P* < 0.05 is considered to be statistically significant. *P < 0.05, **P < 0.01, ***P < 0.001, ****P < 0.0001; Student´s *t* test

Additional analyses of *IGFBP5* expression in both the discovery cohort and the validation cohort revealed an association with lower tumor stage (Fig. 6A). A negative correlation with *PAX3* fusions was noted (Fig. 6B), and when embryonal RMS (eRMS) patients were analyzed separately from alveolar RMS (aRMS), stage 1 tumors demonstrated higher *IGFBP5* levels compared with those > 1 (Fig. 6C). Further support for beneficial properties of *IGFBP5*-expressing tumors was also found in the discovery cohort where *IGFBP5* levels associated with patient survival (Fig. 6D). Similarly, *IGFBP5* associated with favorable overall survival in the validation cohort, while this was not observed in soft-tissue sarcomas with other origin than skeletal muscle (6E).

## Discussion

The mesenchymal identity of sarcomas implies that the malignant cells share many cell-of-origin features with normal mesenchymal stromal cells. It is therefore less clear to what extent the malignant and non-malignant cell compartments complement each other during sarcoma progression similar to what is known from epithelial cancers. To explore this putative discrepancy between mesenchymal and epithelial tumors, our present work aimed to characterize cellular crosstalk between RMS cells and differentially educated stromal cells isolated from skeletal muscle.

Non-malignant mesenchymal stromal cells are known to occur in different cell states and there is no consensus reached on the definition of stromal cells in mesenchymal tumors. For this reason, we aimed to include as many non-malignant mesenchymal stromal cell types, or cell states, as possible in our experimental setup. By immunostaining of tissue sections, both vascular mesenchymal stromal cells (pericytes and smooth muscle cells) and fibroblast-like mesenchymal stromal cells were confirmed to express mouse pdgfrβ (CD140b). Therefore, this pan-marker was the most obvious choice for defining mesenchymal stromal cells throughout the study.

The mesenchymal stromal cell isolation protocol selected for adherent cells from bulk hindlimb muscle. The resulting cell population expressed mouse pdgfrβ and the majority of cells displayed fibroblast morphology. As anticipated, all the primary cell isolations contained proliferating cells and a subpopulation of senescent cells that was not further passaged. For maintained primary cell characteristics of the stromal cells and to avoid unnecessary cellular stress, most analyses relied on paracrine signaling between primary stromal cells and RMS cells under normal growth conditions. Moreover, explorative analysis of data from patient cohorts or human material was always considered a priority when possible.

Initial in vivo experiments demonstrated that the naïve stromal cells isolated from mouse skeletal muscle displayed early tumor growth-suppressive activities in a xenograft model. However, these effects on tumor initiation were impeded as soon as the stromal cells were educated by RMS cells during tumor development in skeletal muscle, or through pre-exposure to tumor cell-derived conditioned medium. For this setup, RD cells were chosen as the best suited model system due to their capacity to engraft both subcutaneously and orthotopically, whereas skeletal muscle was the preferred source for orthotopic stromal cells given the myogenic origin of RMS.

Following cell preparation, ClariomD gene expression analysis identified *IGFBP5* as the most significantly upregulated gene in RMS cells exposed to the naïve mesenchymal stromal cells with tumor growth-suppressive activity. The naïve stromal cells also mediated paracrine *CTGF* downregulation in RD cells, which indicated pro-apoptotic effects, but this was not further dissected. Reassuringly, the candidate gene *IGFBP5* was consistently upregulated in RD cells independently of the anatomic origin or age of the mesenchymal cells from which conditioned medium was collected. And when the experiments were repeated with the RMS cell line RH36, the same pattern of *IGFBP5* upregulation was detected.

A limitation of the study, and the proposed proof-of-principle where naïve stromal cells display transient RMS-suppressive activities, is the heterogeneity of RMS tumors and cell lines with different genetics and phenotypic drivers. IGFBP5 is also a protein with very broad and context-specific functions, which are difficult to fully capture in model systems. Especially ECM composition of representative tumor microenvironments with local enrichment of IGFBP5 is difficult to model correctly. Instead, we analyzed patient material and single cell data to explore the hypothesis that IGFBP5 from neighboring stromal cells and the malignant cells sculptures the tumor microenvironment. The transcriptomics was then accompanied by validation experiments for myogenesis, survival and apoptosis in cell culture using stable cell lines with or without modulated expression.

Previous literature primarily illustrates a role for IGFBP5 in muscle cell differentiation. For instance, *IGFBP5* gene knockdown has been shown to decrease IGF-II expression and lower IGF-IR signaling activity, which led to myogenic differentiation impairment in C2C12 cells in vitro [40]. Given the already reported function of IGFBP5 as a regulator for IGF-1R signaling, particular attention was devoted to differentiation experiments and gene sets for myogenesis. Here, the results showed that IGFBP5 supported myogenic identity of RMS cells in culture as determined by increased *MYF6* expression under regular growth conditions whereas *IGFBP5* knockdown decreased *MYF6* levels. Our study further demonstrated that *IGFBP5* expression levels increased dramatically during myogenic differentiation of cultured RMS cells.

In patient material, the most prominently differentially expressed gene in high versus low *IGFBP5*-expressing tumors was the caspase 3 substrate *GAS2*. When this association was explored in cell culture, IGFBP5 protein treatment increased caspase 3 activity and decreased proliferation of RMS cells. In contrast, *IGFBP5* knockdown decreased caspase 3 activity and increased proliferation. Altogether, this data suggests plausible mechanisms of actions that can explain why *IGFBP5* levels associated with lower tumor stage and favorable survival in the two analyzed patient cohorts.

Noteworthy is that the comparative transcriptomics and single cell data revealed an enrichment for *IGFBP5* in cells with a mesenchymal-like identity, and a more modest expression in muscle, apoptotic cells and proliferative cells sorted from RMS tumors. In normal fibroblasts, expression of *IGFBP5* varied according to their anatomic site of origin. This suggests that even if non-malignant mesenchymal stromal induce *IGFBP5* expression in RMS cells, their own secretion of IGFBP5 may contribute even more to local enrichment of IGFBP5 in the tumor microenvironment. Whether there is a difference between autocrine and paracrine IGFBP5 activities remains to be investigated.

To conclude, our study maps cellular crosstalk between stromal cells and malignant RMS cells and reveals principally different signaling pathways and prognostic biomarkers associated with patient survival. The results further suggest that even if non-malignant mesenchymal stromal cells may initially be tumor growth-suppressive in sarcoma, these activities are easily lost in established tumors. Therefore, to further explore how the tumor-suppressive potential of tissue-resident stromal cells, nearby or at metastatic sites, can be enhanced should be the focus of future studies.

## Conclusions

This study explores anti-tumor growth mechanisms originating from tumor-neighboring mesenchymal stromal cells in RMS, the most common soft-tissue sarcoma in children. Mesenchymal stromal cells belong to a diverse collection of cells in different states that are poorly characterized in non-epithelial tumors, such as sarcomas, and we identify IGFBP5 as an important factor being induced by tumor growth-suppressive mesenchymal stromal cells, and with a capacity to dampen sarcoma cell proliferation, induce caspase 3/7 activity, and support myogenic activity. Altogether, the findings reveal novel mechanisms of actions and encourage further studies on non-malignant stromal cell subsets and paracrine signaling at primary and metastatic sites in malignancies of mesenchymal origin. Future research should focus on translating these findings into clinical applications, potentially leading to innovative treatments that harness the tumor-suppressive properties of mesenchymal stromal cells to improve patient outcomes.

## Supplementary Information

Additional file 1. Control analysis for normalization and removal of batch effects. A. Box plots with all genes, and house-keeping genes only, before and after normalization and removal of batch effects. B. PCA plots with all genes, and house-keeping genes only, after normalization and removal of batch effects.

Additional file 2. Orthotopic naïve stromal cells do not act tumor growth-suppressive in a co-injection xenograft model in mice. Tumor growth of subcutaneous RD xenografts in the presence or absence of co-injected p.SkMSc (20% of total cell number). *N*=9/experimental group. N.S. not significant; Student´s *t* test. Data is presented as the mean ±SD.

Additional file 3. Orthotopic stroma-induced TGFβ signaling associates with patient outcome in soft tissue sarcoma. A. Kaplan-Meier survival analysis of the TGFβ-associated gene signature CCN2/SERPINE1 (CTGF/PAI-1) in the Pan-Can STS cohort. B. Cell survival in the absence or presence of 1.5 µg/ml CTGF protein (ab50044, abcam) under starvation conditions. Total amount of nucleic acid was determined at 72 hours and normalized to the amount of nucleic acid at time 0. *P<0.05; Student´s *t* test. C-D. Volcano plots of genes identified to be differentially expressed in cases with high versus low CCN2 (CTGF) gene expression in the discovery RMS cohort and the validation RMS cohort. Differentially expressed genes are displayed with red dots (absolute value of Log_FC higher than 1.5 and the q-value lower than 0.05) and genes within the gene set Signaling by TGFβ pathway family members are indicated with names if at least one threshold was passed. High versus low gene expression was defined using cohort tertiles.

Additional file 4. Cleaved caspase 3 is detected in the majority of RMS cores. Scoring of cleaved caspase 3 IHC with histoscoring criteria as follows: 0, no positive cells; 1, at least one strongly stained cell; 2, handful strongly stained cells and some weakly stained; 3, at least ten strongly stained cells with preferably nuclear location. Low expression: 0, moderate expression: 1, high expression: 2+3.

Additional file 5. Differentially expressed genes in the IGF1 pathway. A. *IRS1 *(rank 1, q-value <0.001), *PIK3R1* (rank 2, q-value 0.008) and *CSNK2A2* (rank 3, q-value 0.08) were differentially expressed genes comparing high versus low *IGFBP5* gene expression in the Discovery RMS cohort. B. *IGF1* (rank 1, q-value 0.02), *IRS1* (rank 2, q-value 0.009) and *MAP2K1* (rank 3, q-value <0.001) were differentially expressed genes in the Validation RMS cohort comparing high versus low *IGFBP5* gene expression.

Additional file 6. Single cell RNA sequencing reveals prominent IGFBP5 expression in mesenchymal-like cell states in the RMS tumor microenvironment. Three additional patient samples with expression of *IGFBP5* in single cells where cell identity for muscle, apoptosis, proliferation, mesenchymal-like and hypoxia is defined. Investigated genes of interest with a potential link to *IGFBP5* are displayed below and gene expression levels are visualized as percent expressed (size of dots) and average expression (color intensity).

Additional file 7. IGFBP5 levels increase with age and are most prominent in muscle and subsets of fibroblasts. A. *IGFBP5* expression in fetal and adult tissues in normal physiology (visualized in the HTCA database). B. *IGFBP5* expression in adult and fetal cell types in skeletal muscle (visualized in the HTCA database).

Additional file 8. Clariom D gene expression analysis of RD tumor cell response after exposure to tumor growth-suppressive mesenchymal stromal cells. RD tumor cells were exposed to conditioned medium from primary stromal cells isolated from mouse skeletal muscle. Altered gene expression in RD cells was thereafter analysed and top deregulated genes identified.

Additional file 9. Associations of CASP3 expression with disease stage, patient state and translocation.

## Data Availability

Data is provided within the manuscript or supplementary information files.

## Abbreviations

IGFBP: Insulin-like growth factor binding protein 5
RMS: Rhabdomyosarcoma
GAS2: Growth arrest specific protein 2
ECM: Extracellular matrix
PAX: Paired box
FOXO1: (FKHR) forkhead box O1
DDR1: Discoidin domain receptor 1
CTGF: Connective tissue growth factor
PDGF: Platelet-derived growth factor
FGF: Fibroblast growth factor
IGF: Insulin-like growth factor
IGF-1R: Insulin-like growth factor 1 receptor
ITCC: Innovative Therapies for Children and Adolescents with Cancer
DMEM: Dulbecco's Modified Eagle Medium
CCLE: Cancer Cell Line Encyclopedia
TGFβ: Transforming growth factor beta
CTGF: Connective tissue growth factor
PAI-1: Plasminogen activator inhibitor 1
MYF: Myogenic factor
IRS1: Insulin receptor substrate 1
eRMS/aRMS: Embryonal/alveolar rhabdomyosarcoma
PDGFRβ: Platelet-derived growth factor receptor beta
