# Supplementary figures and images for: Myogenic IGFBP5 levels in rhabdomyosarcoma are nourished by mesenchymal stromal cells and regulate growth arrest and apoptosis
Source: Cell Commun Signal. 2025 Apr 15;23:184. doi: 10.1186/s12964-025-02171-6 (PMC12001570; doi:10.1186/s12964-025-02171-6)

Additional file S1

A

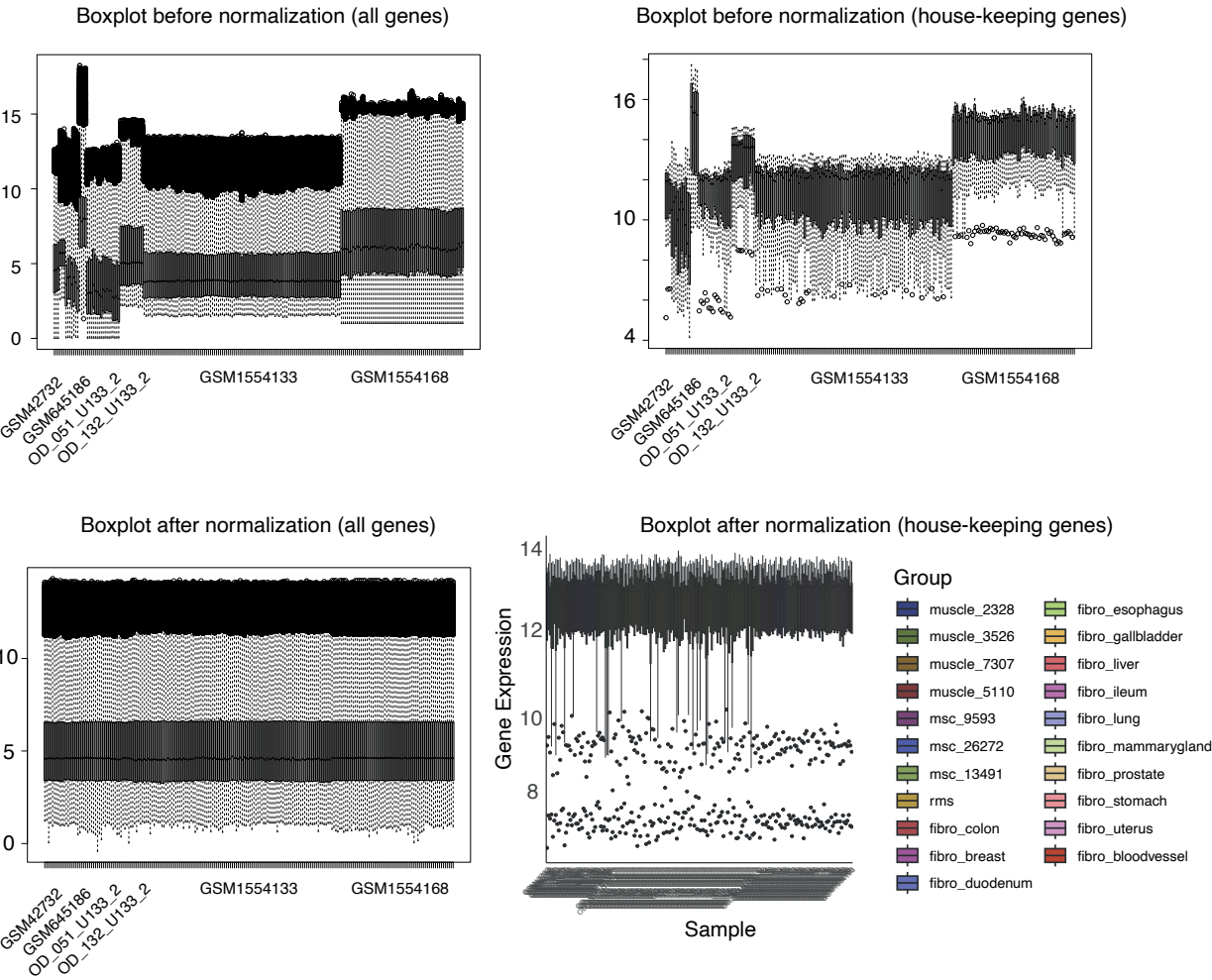

B

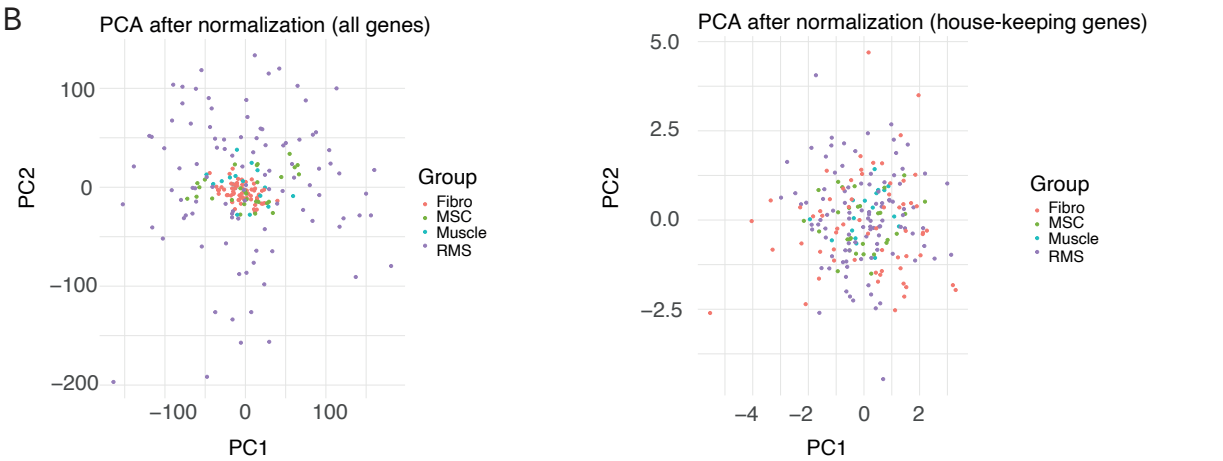

Supplement: Supplementary file 1 — Additional file 1. Control analysis for normalization and removal of batch effects. A. Box plots with all genes, and house-keeping genes only, before and after normalization and removal of batch effects. B. PCA plots with all genes, and house-keeping genes only, after normalization and removal of batch effects. [file 12964_2025_2171_MOESM1_ESM.pdf]

Additional file S2

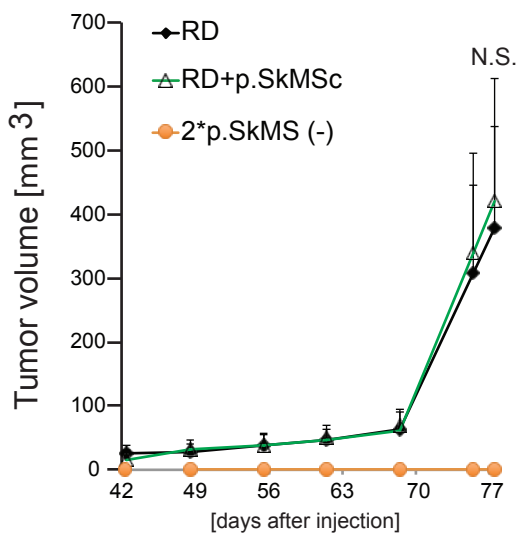

Supplement: Supplementary file 2 — Additional file 2. Orthotopic naïve stromal cells do not act tumor growth-suppressive in a co-injection xenograft model in mice. Tumor growth of subcutaneous RD xenografts in the presence or absence of co-injected p.SkMSc (20% of total cell number). N=9/experimental group. N.S. not significant; Student´s t test. Data is presented as the mean ±SD. [file 12964_2025_2171_MOESM2_ESM.pdf]

Additional file S4

Cleaved caspase 3 expression

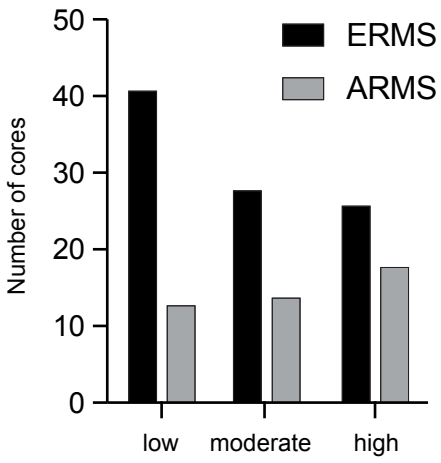

Supplement: Supplementary file 4 — Additional file 4. Cleaved caspase 3 is detected in the majority of RMS cores. Scoring of cleaved caspase 3 IHC with histoscoring criteria as follows: 0, no positive cells; 1, at least one strongly stained cell; 2, handful strongly stained cells and some weakly stained; 3, at least ten strongly stained cells with preferably nuclear location. Low expression: 0, moderate expression: 1, high expression: 2+3. [file 12964_2025_2171_MOESM4_ESM.pdf]

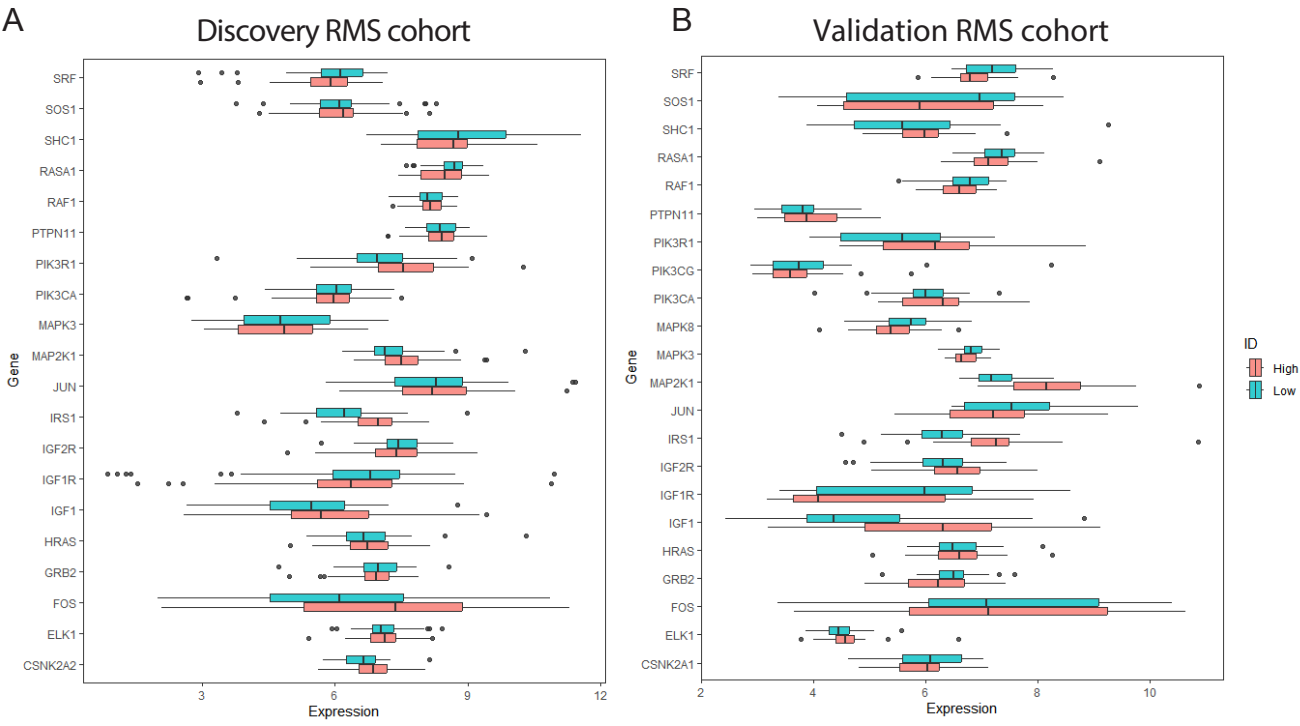

Supplement: Supplementary file 5 — Additional file 5. Differentially expressed genes in the IGF1 pathway. A. IRS1 (rank 1, q-value <0.001), PIK3R1 (rank 2, q-value 0.008) and CSNK2A2 (rank 3, q-value 0.08) were differentially expressed genes comparing high versus low IGFBP5 gene expression in the Discovery RMS cohort. B. IGF1 (rank 1, q-value 0.02), IRS1 (rank 2, q-value 0.009) and MAP2K1 (rank 3, q-value <0.001) were differentially expressed genes in the Validation RMS cohort comparing high versus low IGFBP5 gene expression. [file 12964_2025_2171_MOESM5_ESM.pdf]

Additional file S6

Patient A

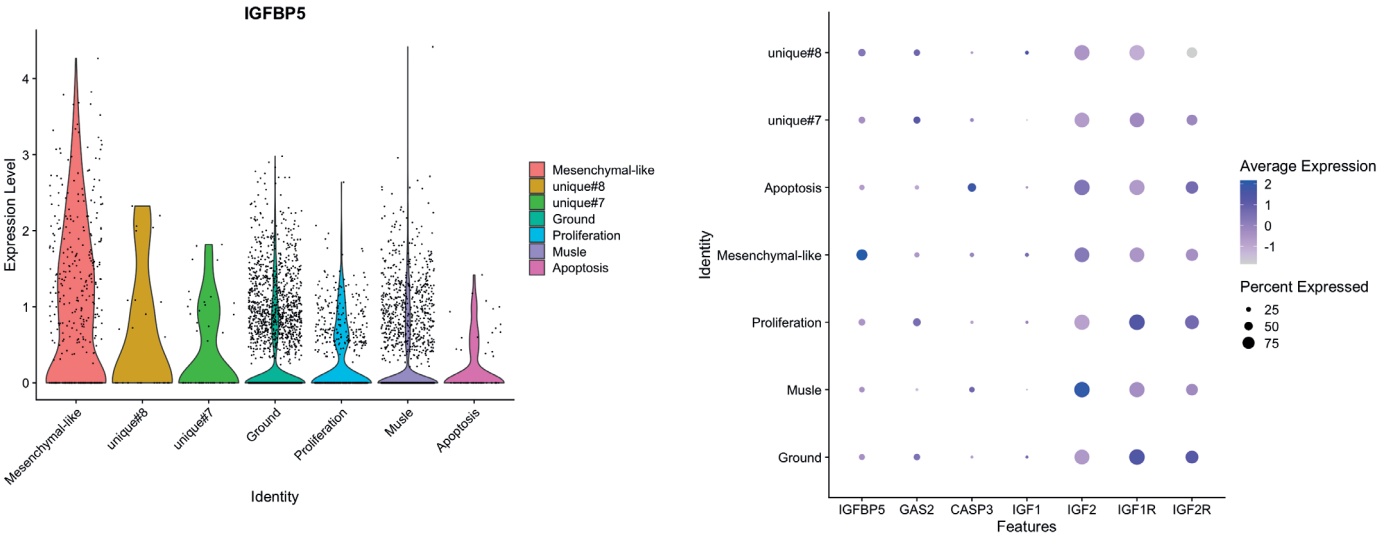

Patient B

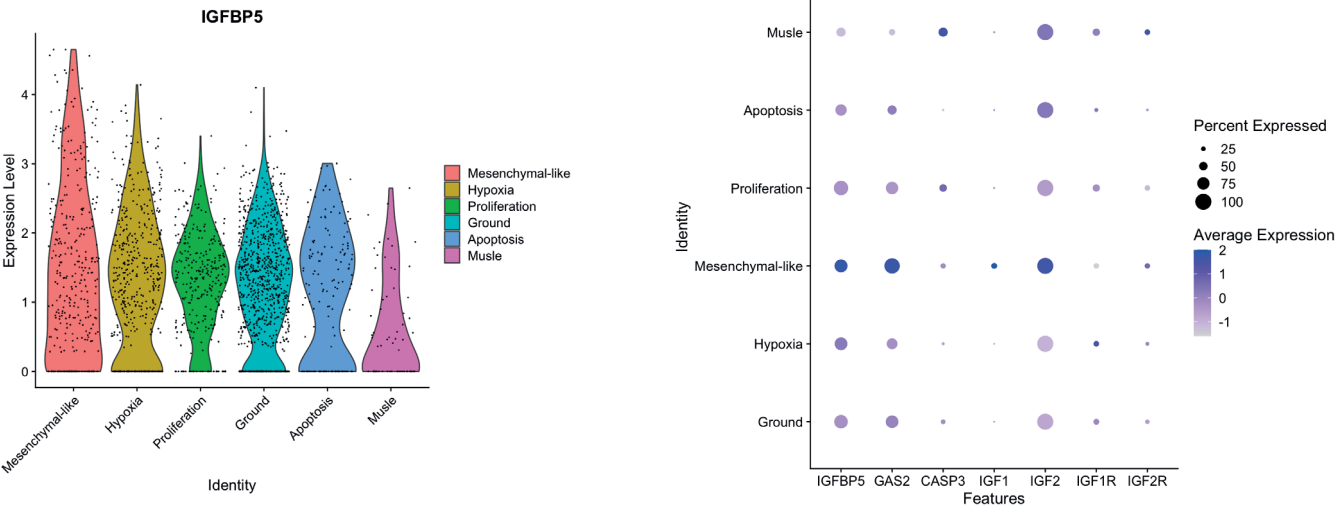

Patient C

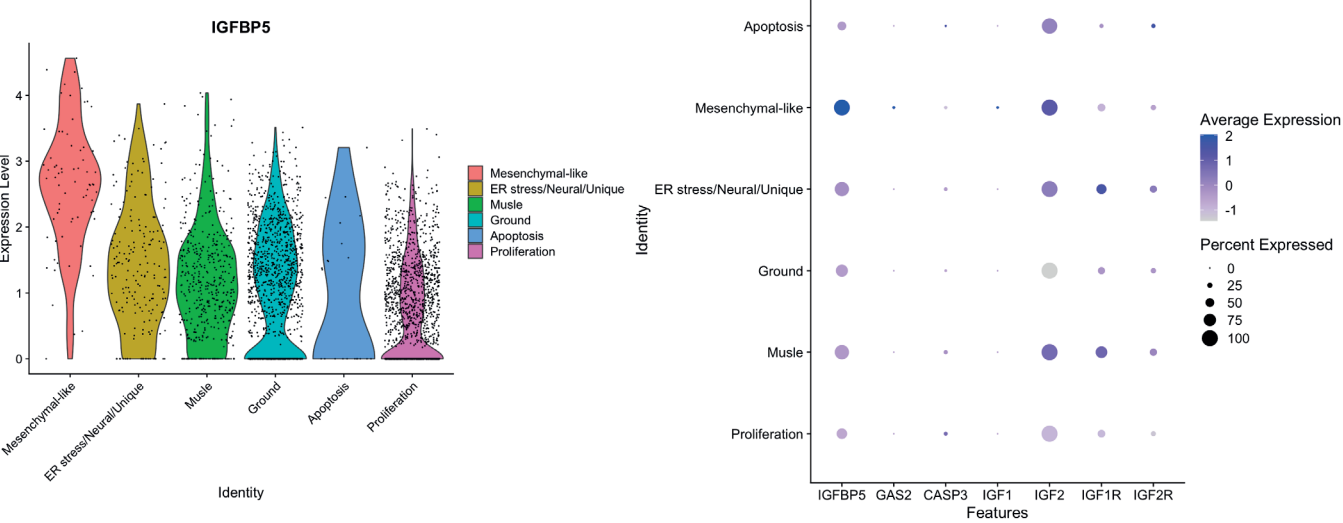

Supplement: Supplementary file 6 — Additional file 6. Single cell RNA sequencing reveals prominent IGFBP5 expression in mesenchymal-like cell states in the RMS tumor microenvironment. Three additional patient samples with expression of IGFBP5 in single cells where cell identity for muscle, apoptosis, proliferation, mesenchymal-like and hypoxia is defined. Investigated genes of interest with a potential link to IGFBP5 are displayed below and gene expression levels are visualized as percent expressed (size of dots) and average expression (color intensity). [file 12964_2025_2171_MOESM6_ESM.pdf]

A

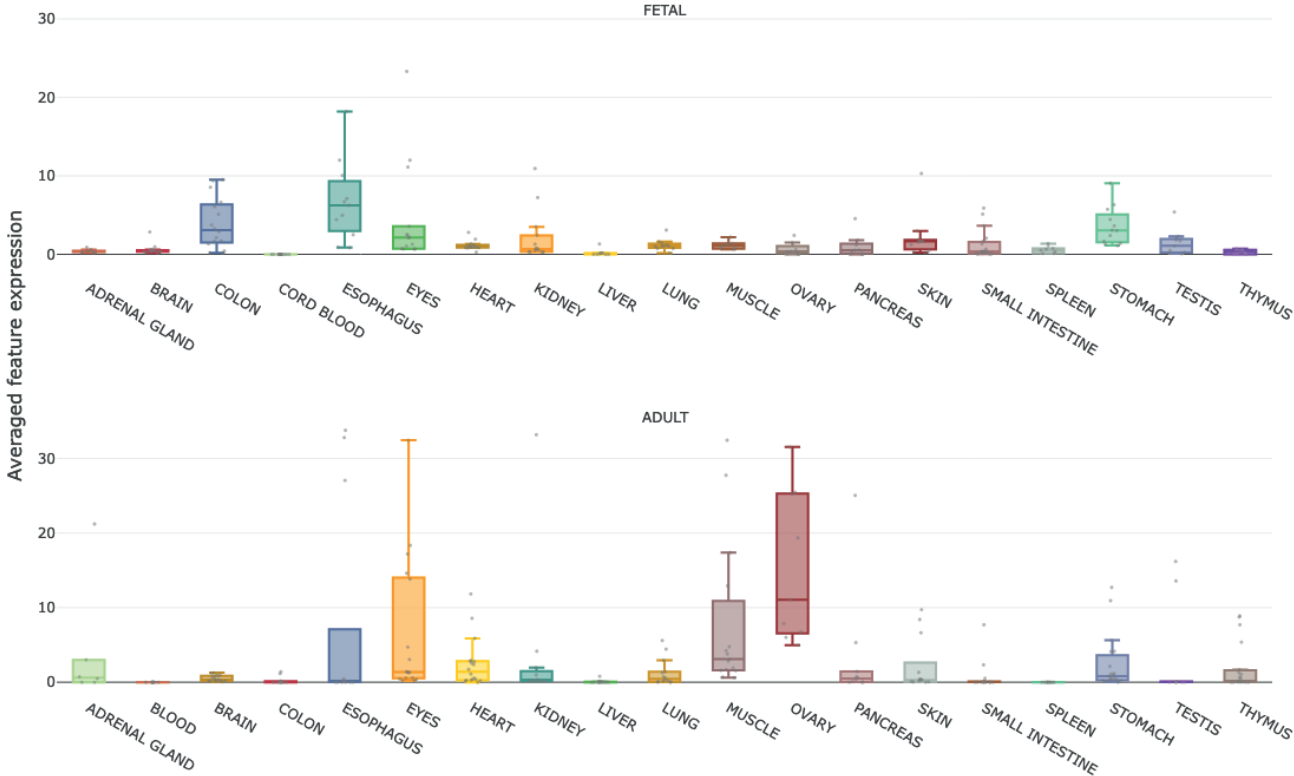

B

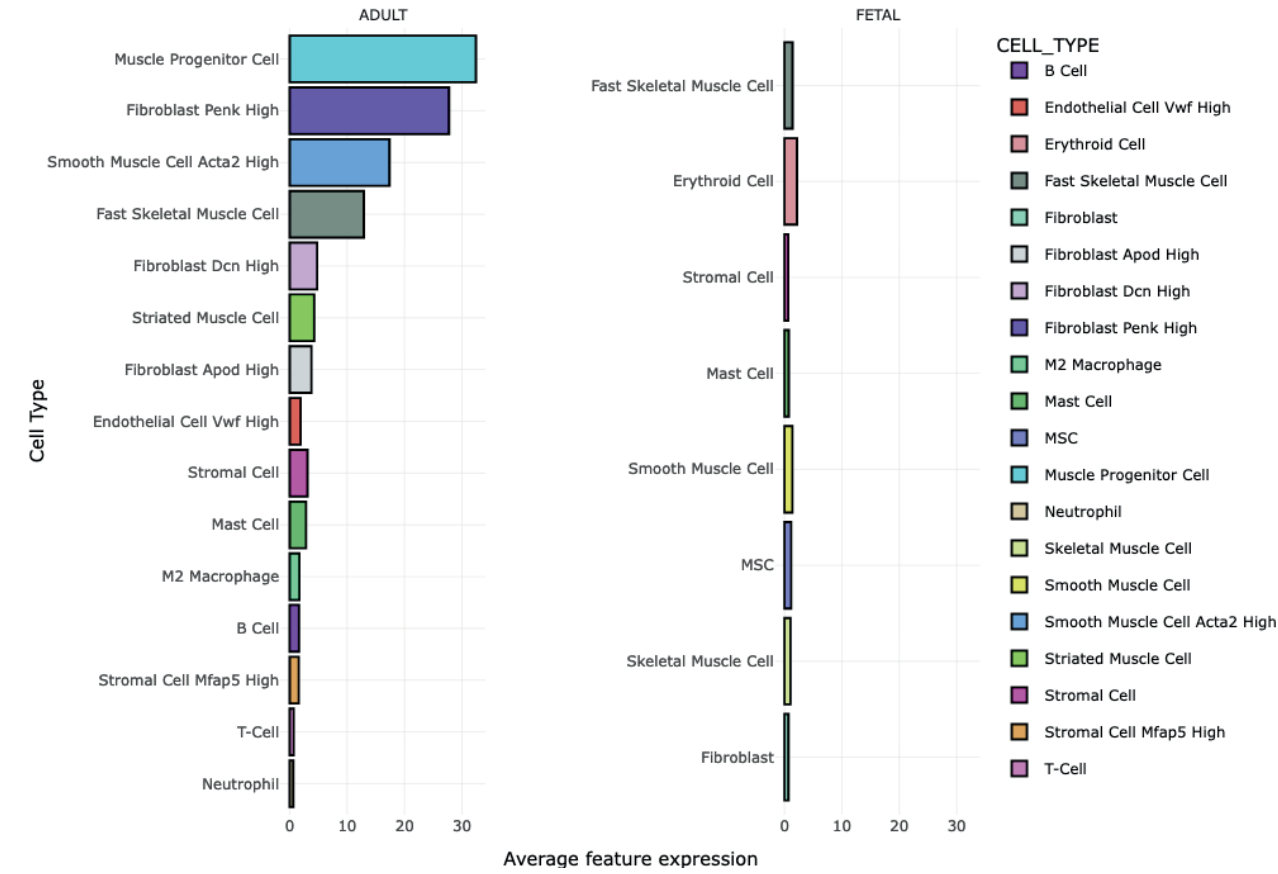

Supplement: Supplementary file 7 — Additional file 7. IGFBP5 levels increase with age and are most prominent in muscle and subsets of fibroblasts. A. IGFBP5 expression in fetal and adult tissues in normal physiology (visualized in the HTCA database). B. IGFBP5 expression in adult and fetal cell types in skeletal muscle (visualized in the HTCA database). [file 12964_2025_2171_MOESM7_ESM.pdf]
