# Supplementary material for: Myogenic IGFBP5 levels in rhabdomyosarcoma are nourished by mesenchymal stromal cells and regulate growth arrest and apoptosis
Source: Cell Commun Signal. 2025 Apr 15;23:184. doi: 10.1186/s12964-025-02171-6 (PMC12001570; doi:10.1186/s12964-025-02171-6)
Supplement: Supplementary file 8 — Additional file 8. Clariom D gene expression analysis of RD tumor cell response after exposure to tumor growth-suppressive mesenchymal stromal cells. RD tumor cells were exposed to conditioned medium from primary stromal cells isolated from mouse skeletal muscle. Altered gene expression in RD cells was thereafter analysed and top deregulated genes identified. [file 12964_2025_2171_MOESM8_ESM.docx]

| **Top deregulated genes** | **Fold change** | **p**  **value** | **q value** |
| --- | --- | --- | --- |
| 1. UNC80 | -4.8 | 0.00 | 0.45 |
| 2. MIR548AI | -3.0 | 0.22 | 0.90 |
| 3. CTGF/CCN2 | -2.5 | 0.00 | 0.65 |
| 4. LINC00702 | -2.2 | 0.01 | 0.90 |
| 5. LOC101927229 | -2.2 | 0.00 | 0.75 |
| 6. SCARNA23 | -2.0 | 0.12 | 0.90 |
| 7. KLHL41 | -2.0 | 0.00 | 0.40 |
| 8. LYPD1 | -2.0 | 0.01 | 0.85 |
| 9. SERPINE1/PAI-1 | -2.0 | 0.00 | 0.40 |
|  |  |  |  |
| 1. IGFBP5 | 11.9 | 0.00 | 0.40 |
| 2. MFAP4 | 3.2 | 0.00 | 0.40 |
| 3. SULF2 | 2.4 | 0.00 | 0.47 |
| 4. SPOCK2/SPARC | 2.3 | 0.00 | 0.40 |
| 5. PDE4B | 2.3 | 0.00 | 0.40 |
| 6. IGFBP3 | 2.3 | 0.00 | 0.40 |

**Additional file, Table 1. Clariom D gene expression analysis of RD tumor cell response after exposure to tumor growth-suppressive mesenchymal stromal cells.** RD tumor cells were exposed to conditioned medium from primary stromal cells isolated from mouse skeletal muscle. Altered gene expression in RD cells was thereafter analysed and top deregulated genes identified.
