# Supplementary material for: Myogenic IGFBP5 levels in rhabdomyosarcoma are nourished by mesenchymal stromal cells and regulate growth arrest and apoptosis
Source: Cell Commun Signal. 2025 Apr 15;23:184. doi: 10.1186/s12964-025-02171-6 (PMC12001570; doi:10.1186/s12964-025-02171-6)
Supplement: Supplementary file 9 — Additional file 9. Associations of CASP3 expression with disease stage, patient state and translocation. [file 12964_2025_2171_MOESM9_ESM.docx]

**Additional file, Table 2. Associations of CASP3 expression with disease stage, patient state and translocation.**

| CASP3 in discovery RMS cohort | | | | | | |  | CASP3 in validation RMS cohort | | | | | | |
| --- | --- | --- | --- | --- | --- | --- | --- | --- | --- | --- | --- | --- | --- | --- |
| Group | Disease stage | | Patient state | | Translocation | |  | Group | Disease stage | | Patient state | | Translocation | |
|  | Stage 1 | Stage 1+ | Alive | Dead | PAX3 | Neg |  |  | Stage 1 | Stage 1+ | Alive | Dead | PAX3 | Neg |
| High | 7 | 34 | 31 | 18 | 7 | 25 |  | High | 10 | 24 | 25 | 9 | 7 | 26 |
| Moderate | 12 | 27 | 31 | 15 | 14 | 18 |  | Moderate | 12 | 21 | 19 | 14 | 16 | 15 |
| Low | 5 | 39 | 34 | 15 | 17 | 15 |  | Low | 6 | 27 | 23 | 11 | 11 | 15 |
|  | χ2 5.19; *P* = 0.075 | | χ2 0.429; *P* = 0.81 | | χ2 6.88; *P* = 0.032 | |  |  | χ2 2.76; *P* = 0.252 | | χ2 1.95; *P* = 0.38 | | χ2 6.60; *P =* 0.037 | |
|  |  |  |  |  |  |  |  |  |  |  |  |  |  |  |
|  |  |  |  |  |  |  |  |  |  |  |  |  |  |  |
